# Comparison of Biomolecular Condensate Localization and Protein Phase Separation Predictors

**DOI:** 10.3390/biom13030527

**Published:** 2023-03-13

**Authors:** Erich R. Kuechler, Alex Huang, Jennifer M. Bui, Thibault Mayor, Jörg Gsponer

**Affiliations:** Michael Smith Laboratories, Department of Biochemistry and Molecular Biology, University of British Columbia, Vancouver, BC V6T 1Z4, Canada

**Keywords:** protein phase separation, intrinsically disordered proteins, prediction methods

## Abstract

Research in the field of biochemistry and cellular biology has entered a new phase due to the discovery of phase separation driving the formation of biomolecular condensates, or membraneless organelles, in cells. The implications of this novel principle of cellular organization are vast and can be applied at multiple scales, spawning exciting research questions in numerous directions. Of fundamental importance are the molecular mechanisms that underly biomolecular condensate formation within cells and whether insights gained into these mechanisms provide a gateway for accurate predictions of protein phase behavior. Within the last six years, a significant number of predictors for protein phase separation and condensate localization have emerged. Herein, we compare a collection of state-of-the-art predictors on different tasks related to protein phase behavior. We show that the tested methods achieve high AUCs in the identification of biomolecular condensate drivers and scaffolds, as well as in the identification of proteins able to phase separate in vitro. However, our benchmark tests reveal that their performance is poorer when used to predict protein segments that are involved in phase separation or to classify amino acid substitutions as phase-separation-promoting or -inhibiting mutations. Our results suggest that the phenomenological approach used by most predictors is insufficient to fully grasp the complexity of the phenomenon within biological contexts and make reliable predictions related to protein phase behavior at the residue level.

## 1. Introduction

Protein interactions with other macromolecules form the backbone of cellular organization and communication [1,2]. While most protein interactions involve stochiometric complexes, it has come to light that non-stochiometric, often dynamic protein interactions underline the formation of biomolecular condensates [3,4,5,6]. These membraneless organelles form through a process known as phase separation and play vital roles in cellular systems [7,8,9,10]. These assemblies contain protein and nucleic acid constituents that are in fast exchange with the surrounding and, thus, display a liquid-like character and viscoelastic properties distinct from the immediate environment [11]. The discovery of protein phase separation as a driving principle for cellular organization at multiple scales has not only advanced our understanding of biology but also significantly changed our scientific endeavors.

To further understand how biomolecular condensates contribute to cellular organization and function, it is crucial to assemble a complete map of the “granulome”, i.e., the list of all proteins that participate in membraneless organelles. Seminal work over the past decade has charted the membership of numerous proteins within a variety of biological condensates utilizing SILAC (stable isotope labeling with amino acids in cell culture) [12], proximity-labelling-based mass spectrometry approaches [13,14], and other methods [15,16,17]. Additionally, there has been a concerted effort in the community to catalog the granulome members [18,19,20,21]. Significant strides have also been made to best determine which proteins are capable of undergoing phase separation by themselves in vitro [9,22,23]. Proteins that are capable of forming droplets in vitro and are necessary for biomolecular condensate formation are commonly referred to as protein ‘drivers’ or ‘scaffolds’, while other granulome proteins that do not possess the proclivity to phase separate by themselves are referred to as ‘clients’ [24]. It is commonly thought that these clients enter into a phase separated compartment by interacting with one or more drivers; then, clients are shuttled into condensates via their interaction partners. However, it should be noted that numerous clients have not been extensively characterized and/or individually tested in vitro, and more scaffold proteins may exist. This differentiation in class of granulome proteins highlights a key challenge in predicting whether a protein is likely to localize to a membraneless organelle within the cell. While all granulome proteins may share a set of common features, protein features for clients and scaffolds are likely to be different.

It needs to be stressed that the biophysical principles that govern protein phase separation are well-understood [6,25]. Given specific boundary conditions (T, pH and solvent composition), certain proteins prefer, beyond a concentration threshold, to interact with themselves (simple coacervation) or with other proteins (complex coacervation) than with the solvent. Which type of molecular interactions are favored is often less clear. From the wealth of available data, it is, at least, evident that multiple interactions need to be involved; proteins that phase separate are multi-valent. To this end, the leading hypothesis on protein phase separation promotes the associative polymer idea, according to which the proteins contain ”sticker” and “spacer” elements [26]. Interacting protein parts are designated as stickers that are connected by flexible spacers. The composition and architecture of sticker regions can vary drastically, from structured domains to molecular recognition features (MoRFs), short interaction motifs or individual amino acids in intrinsically disordered protein parts [25,27]. This variety in interaction types involving only one or combinations of proteins (or even nucleic acids) poses a significant challenge in cataloging a precise list of important interaction modalities for phase separation and biomolecular condensate localization. Despite this hurdle and other challenges, several predictors of granule localization and/or phase separation have been developed to aid the experimental discovery of these proteins [28,29,30,31,32,33,34,35,36,37,38]. These predictors leverage a wide variety of approaches, from trying to understand the molecular mechanisms that cause proteins to phase separate and localize to biomolecular condensates, to predictions that are primarily phenomenologically based.

Herein, we examine the current state of computational predictors that are publicly available. We test both type of predictors: those that have been explicitly trained to predict biomolecular condensate localization and phase separation, respectively. Specifically, we test them for their ability to predict biomolecular condensate localization, in vitro phase separation, regions that are involved in phase separation and the impact of sequence variation on phase separation.

## 2. Materials and Methods

Datasets for human phase separating proteins were curated from the DrLLPS [18], PhaSepDB2.1 [20,21], and PhaSePro [19] databases, as well as using training data from publications of phase separation predictors. We selected four different condensates for which there are large numbers of components identified. In total, we assembled 1399 stress granule proteins, 2396 nucleolar proteins, 497 p-body proteins and 142 nuclear speckle proteins. To identify scaffolds for each type of biomolecular condensate, we matched these sets with 89 verified scaffold proteins obtained from the work of Farahi et al. [39]. Additionally, we assembled 132 proteins that have experimental evidence for phase separation in vitro. In this scheme, there are some overlaps between the datasets but also clear distinctions. We created two types of negative sets to calculate ROC curves: (NS1) a set created from proteins in the human proteome that are not listed in the condensate databases considered here, and (NS2) a set of single domain PDB proteins that was used as a negative control in the work conducted by Cai et al. [36], with the exception of any proteins found in the listed condensate databases. To examine the performance of sequence-based predictors we created a set of proteins with known regions that have been observed to undergo homotypic phase separation or being necessary for proteins to localize to condensates. Specifically, residues in regions that have been observed to undergo homotypic phase separation or being necessary or sufficient for a protein being included in phase separated droplets were used in the positive set PR. All residues in other regions in the same proteins served as negative set (NR). Using this method, we assembled a set of 11,742 phase separation positive and 20,884 phase separation negative residues over 46 proteins. All positive datasets used in this study can be found in Supplementary Table S1.

The methods tested in this manuscript include: PScore [28], PSPredictor [29], FuzDrop [31], catGranule [32], PSAP [33], PhaSePred [34], DeePhase [35], LLPhyScore [36], ParSe [37], MaGS [38], and MaGSeq [30]. Here, we provide a brief description of each of the methods:

- PScore estimates phase separation proclivity based on pi-interaction frequency under the assumption that cation–pi, pi–pi, and charge interactions promote a multivalent behavior. This machine learning model was trained to predict these interactions using over 10,000 structures from the PDB (Protein Data Bank).
- PSPredictor uses evolutionary word2vec sequence encoding with gradient boosting decision tree machine leaning algorithms to generate a prediction model that differentiates phase separating proteins using the LLPSDB dataset.
- Fuzdrop predicts droplet-promoting regions by assuming that the large conformational entropy associated with nonspecific side–chain interactions is the driving force of droplet inclusion. This model was trained on a wide range of data including in vitro and in vivo annotated membraneless organelle proteins.
- catGranule is a linear model trained on 120 granule-forming proteins from the yeast proteome. Protein features in this model largely focus on RNA-binding, protein disorder, and amino acid composition.
- PSAP uses a random forest approach to predict the probability of proteins to undergo phase separation and was trained on 90 high-confidence human phase separating proteins that act as drivers.
- PhaSePred is a meta-predictor that utilizes a XGB16oost tree model to distinguish phase separating proteins and non-phase separating controls. It was trained on 658 experimentally validated phase separating proteins across the PhaSepDB, LLPSDB, and PhaSePro databases. In the current study, we used the eight-feature sum for the generation of our data.
- DeePhase predictor of homotypic liquid–liquid phase separation identifies proteins by combining knowledge-based features with unsupervised embeddings from a pretrained word2vec model using 3-g as words and a context window size of 25.
- LLPhyScore was trained on validated phase separating proteins from the PhaSepDB, LLPSDB, and PhaSePro databases and uses 16 weighted features, including pi–pi and charge interactions.
- ParSe estimates phase separation likelihood by combining metrics for the hydrodynamic size of monomeric proteins with protein’s sequence-predicted propensity for β-turns. This model was parameterized using phase separating proteins that were collected from the PhaSePro and DISPROT databases as well as from the literature.
- MaGS and MaGSeq use enriched protein features of granule proteins to generate linear models for protein condensate localization prediction. These models were parameterized using client and scaffold proteins of stress granules listed in the DrLLPS database.

Additionally, a number of disorder predictors were used for control comparisons in this study, which included DISOPRED3 [40], IUPred2 [41,42], MetaPredict [43], and DISPROT [44]. Further, the sequence-based RNA-binding predictor RBPPred [45] was used to control for this leading feature of granule proteins. These controls were established to determine whether tested models are mainly recapitulating one of these fundamental features.

Differences between prediction scores of wild type and mutated sequences, the prediction delta scores, were used to impute the impact of mutations on phase behavior. Specifically, a mean delta score was calculated for scores falling within an appropriate window size of the point mutation sites. This was necessary because the tested predictors use running windows to generate their prediction scores for individual residues. The mean near-site delta was normalized by the standard error using the variance of delta scores across the entire protein. Treating the normalized near-site delta score as a Z-score, we interpreted scores above the 95% confidence interval (CI) as predicting a phase separation promoting mutation, and scores below the 95% CI as predicting a phase separation inhibitory mutation. In detail, scores were calculated as:

$$\Delta_{\mathrm{mt}}\left(x\right)=s_{mt}\left(x\right)-s_{wt}\left(x\right)$$

σmt=∑x[Δmtx−∑xΔmtxntotal]^2ntotal−1

$${z\Delta }_{\mathrm{mt}}=\left[\frac{\sum_{x\;\mathrm{near}\;\mathrm{site}}\Delta_{mt}\left(x\right)}{\sigma_{mt}\sqrt{n_{\mathrm{near}\;\mathrm{site}}}}\right]$$

Statistics and analyses were performed using the R statistical package (v4.2.1) [46] and data curation was performed using in-house Perl (v5.30.0) scripts [47]. Graphics were generated using base R packages and ggplot2 [48]. Graphics were then combined into total figures using Adobe Illustrator.

## 3. Results

### 3.1. Prediction of Biomolecular Condensate Localization

To compare the performance of existing predictors, we assembled datasets of proteins that localize into biomolecular condensates in human cells. To this end, we collected data from a number of databases and across the literature, the specific numbers of which can be found in the Methods section. We assembled two distinct negative control sets: (i) all proteins from the human proteome that are not in the combined positive sets (NS1); and (ii) single domain proteins with PDB structures that are not in the combined positive sets (NS2). The latter negative set was selected because phase separating proteins are often multi-valent. Proteins that have well-defined structures and only a single domain are less likely to be capable of forming these types of interactions. While phase separation can also be mediated by multiple discrete interaction patches on single domain proteins, similar control sets have been used to parameterize deep learning models for phase separation due to the likelihood of containing fewer false negatives than NS1.

**Figure 1:**
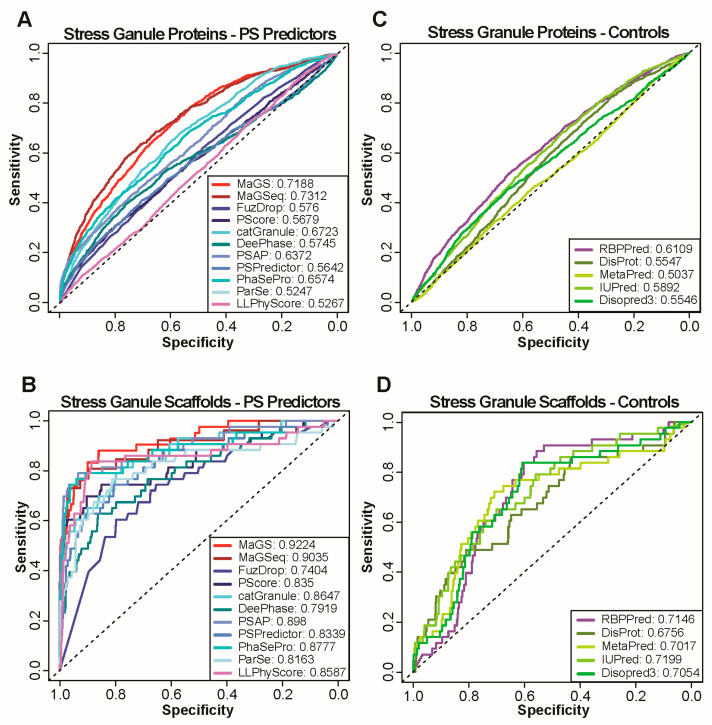
Performance of state-of-the art methods and simple protein features on stress granule localization prediction. (**A**,**C**) ROC curves calculated with predictions by catGranule, DeepPhase, FuzDrop, MaGS, MaGSeq, ParSe, PSAP, PScore, PSPred and LLPhyScore (**A**) as well as simple protein features obtained by using the sources listed (**C**). Curves are calculated using all stress granule proteins as positives and NS1 as negative set. AUC values are provided for each method in the legend. (**B**,**D**) ROC curves calculated with predictions of same methods and features as in A and C. Curves are calculated using only scaffolds of stress granule formation as positives and NS1 as negative set.

We first focused our analysis on proteins that localize in cytosolic stress granules, foci that form when a cell is under external threat, as these membraneless compartments are among the most studied biomolecular condensates. To test the performance of multiple predictors, we generated receiver operating characteristic (ROC) curves and calculated the area under the ROC curves (AUC) for the prediction of stress granule localization for catGranule, DeePhase, FuzDrop, MaGS, MaGSeq, ParSe, PSAP, PhaSePred, PScore, PSPredictor, and LLPhyScore using each of the two negative sets (Figure 1A and Supplementary Figure S1A,B).

The AUCs reveal modest performances of all predictors on stress granule localization, with MaGSeq displaying the best performance (AUC: 0.73). We next assessed proteins localized in other well-characterized biomolecular condensates. Notably, we selected cytosolic p-bodies that are constitutively present in cells and also share constituents with stress granules [16], the nucleolus that forms a multiphase condensate in which ribosomes are assembled [49], and nuclear speckles that are enriched with mRNA splicing factors [50]. Similar to the predictions for stress granules, we obtained relatively low performances (Supplementary Figure S1C–H), with the exception of nuclear speckles for which AUCs of up to 0.84 are achieved using MaGSeq.

A large number, if not most, of the biomolecular condensate members locate there as clients and not as scaffolds or drivers of condensate formation. As most predictors are trained to identify phase separating proteins, a more appropriate prediction evaluation should focus on scaffold/drivers only. Therefore, we focused on a subset of proteins shown to be scaffolds (see Methods). On datasets of scaffolds of condensate formation, all predictors perform better than on the full condensate sets (Figure 1B and Supplementary Figure S2A–D, only results using NS1 are shown). On scaffolds of stress granules (Figure 1B), most predictors achieve AUCs above 0.8, with the highest performance being realized by MaGS (AUC:0.92). Similarly high AUCs are achieved for scaffolds of other granules (Supplementary Figure S2A–D).

### 3.2. Prediction of In Vitro Phase Separation

**Figure 2:**
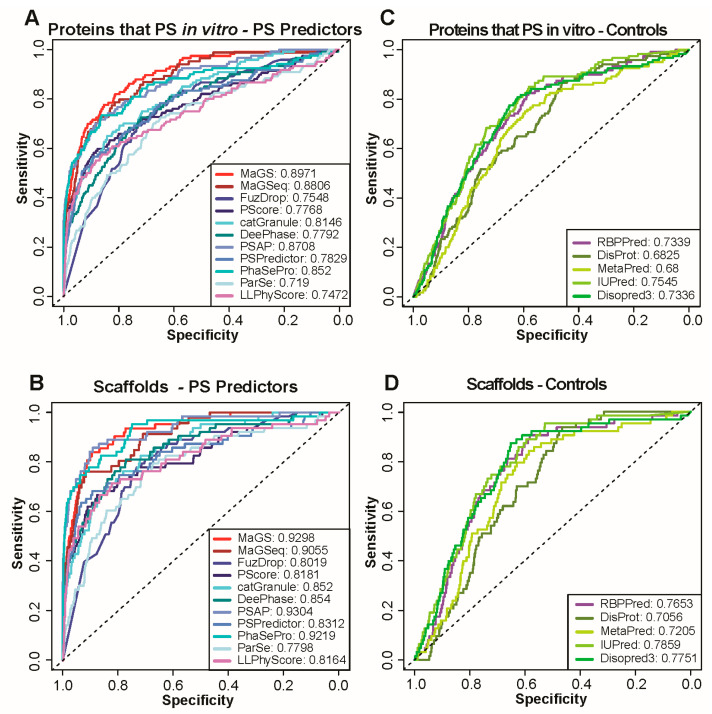
Performance of state-of-the-art methods and simple protein features for in vitro phase separation (PS) prediction. (**A**,**C**) ROC curves calculated with predictions of the same methods used for Figure 1 (**A**) as well as simple protein features obtained by using the sources listed (**C**). Curves are calculated using proteins known to phase separate in vitro as positives and NS1 as negative set. AUC values are provided for each method in the legend. (**B**,**D**) ROC curves calculated with predictions of the same methods and features as in A and C. Curves are calculated using only scaffolds as positives and NS1 as negative set.

in vitro studies of protein phase separation currently provide most of the molecular insights into the driving forces behind the process. As a result, most predictors relied on and exploited in vitro data during training and are well-optimized to predict proteins that undergo in vitro phase separation. To test the performance of predictors on this subset of proteins, we selected an additional positive set that consists of proteins annotated as those that undergo phase separation in vitro in the aforementioned databases. NS1 and NS2 served again as negative sets. As expected, all predictors perform much better in the identification of these proteins compared to those that localize within condensates (Figure 2A and Supplementary Figure S3). The highest AUCs for in vitro phase separation prediction are achieved by MaGS, MaGSeq and PSAP, independent of the negative set used. The performance of most predictors, in terms of AUC, improves when only the subset of phase separating proteins that is taken is also known to be scaffolds of biomolecular condensates (Figure 2B, only results using NS1 are shown).

### 3.3. Prediction Performance of Simple Protein Features

The biophysical principles that govern protein phase separation are well-understood. However, the molecular details of the interactions that are present in specific condensates remain more elusive and have only been mapped in specific cases [51,52,53,54]. Most predictors therefore exploit generic features that have been found enriched among proteins that phase separate. Most prominent among these features are an elevated amount of intrinsic protein disorder and a proteins’ ability to bind RNA. The former is a feature that is associated with many proteins that were initially found to phase separate, specifically proteins that have prion-like domains [55,56,57]. The latter is a feature associated with many proteins that are members of biomolecular condensates in cells, specifically those proteins that localize to the arguably most studied cellular condensates, stress granules and P-bodies.

Given the strong association of these two features with biomolecular condensate localization in cells and in vitro phase separation, we decided to evaluate the predictive power of these two features alone. To this end, we tested the performance of the disorder predictors Disopred3, IUpred2, DisProt, and MetaPredict on the same tasks as the phase separation predictors. A variety of predictors were used to alleviate any concerns in bias for a given method. For RNA-binding propensity, we used the RBPPred software. The performance of these methods on the prediction of biomolecular condensate localization is, in many cases, relatively high given that they were not trained to recapitulate this behavior. The RNA-binding feature alone achieves AUCs that come close to or even exceed those of several existing predictors (Figure 1C and Supplementary Figure S1), which is particularly evident for stress granules or P-bodies membership predictions. Importantly, RNA-binding predicts biomolecular condensate localization better than a protein’s intrinsic disorder for all tested granules with the exception of nuclear speckles. The results change when only established scaffolds of condensate formation are used in positive test sets (Figure 1D and Supplementary Figure S2). Most dedicated phase separation predictors separate scaffolds from proteins not known to be scaffolds better than the RNA-binding feature or a protein’s intrinsic disorder alone. This said, the RNA-binding predictor continues to outperform several phase separation predictors for nuclear speckles scaffolds.

As far as in vitro phase separation is concerned, a protein’s intrinsic disorder predicted via IUPred2 is more efficient in separating positive and negative test sets than RNA-binding (Figure 2C and Supplementary Figure S3). More importantly, the AUCs calculated with intrinsic protein disorder scores of IUPred2 and Disopred3 come close to or even exceed the AUC of some tested predictors. This finding changes only slightly when the positive set consists only of the subset of phase separating proteins that are also scaffolds of biomolecular condensates in cells (Figure 2D).

### 3.4. Prediction of Protein Segments That Are Involved in Phase Separation

**Figure 3:**
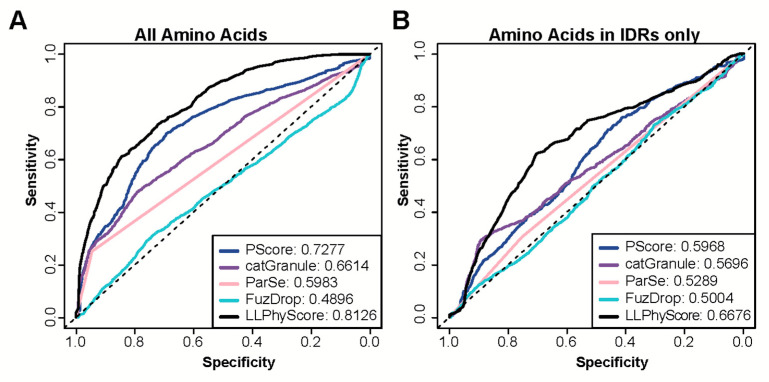
Performance of state-of-the art methods on residue-level prediction of segments involved in phase separation. (**A**,**B**) ROC curves calculated with predictions by catGranule, FuzDrop, Parse, PScore and LLPhyScore. AUC values are provided for each method in the legend. (**A**) Curves are calculated using all residues in protein segments identified experimentally to be involved in phase separation as positives (PR) and all remaining residues of the same proteins as negatives (NR). (**B**) Curves are calculated using only those residues in PR and NR that are part of predicted intrinsically disordered regions (IDRs).

In addition to providing a protein-level score that can be used to assess the probability of a protein to phase separate or localize to biomolecular condensates, some of the recently available predictors also provide scores at the residue level. Such residue scores are helpful in the identification of segments in proteins that are likely driving the phase separation process or are at least involved in interactions that are necessary for phase separation. Therefore, we next tested the ability of these predictors to correctly identify these segments. To this end, we collected a set of proteins with known regions that have been observed to undergo homotypic phase separation or being necessary or sufficient for a protein being included in phase separated droplets (positive set PR). All other regions in the same proteins served as negative set (NR). We then used a series of predictors with the capability to output residue-level annotations (PScore, LLPhyScore, catGranule, FuzDrop and ParSe) to evaluate whether predictions were specific enough to recapitulate experimental findings. Only LLPhyScore and PScore achieve high AUCs in this prediction task (Figure 3A), which may not be surprising as both methods exploit molecular level information for their predictions.

**Figure 4:**
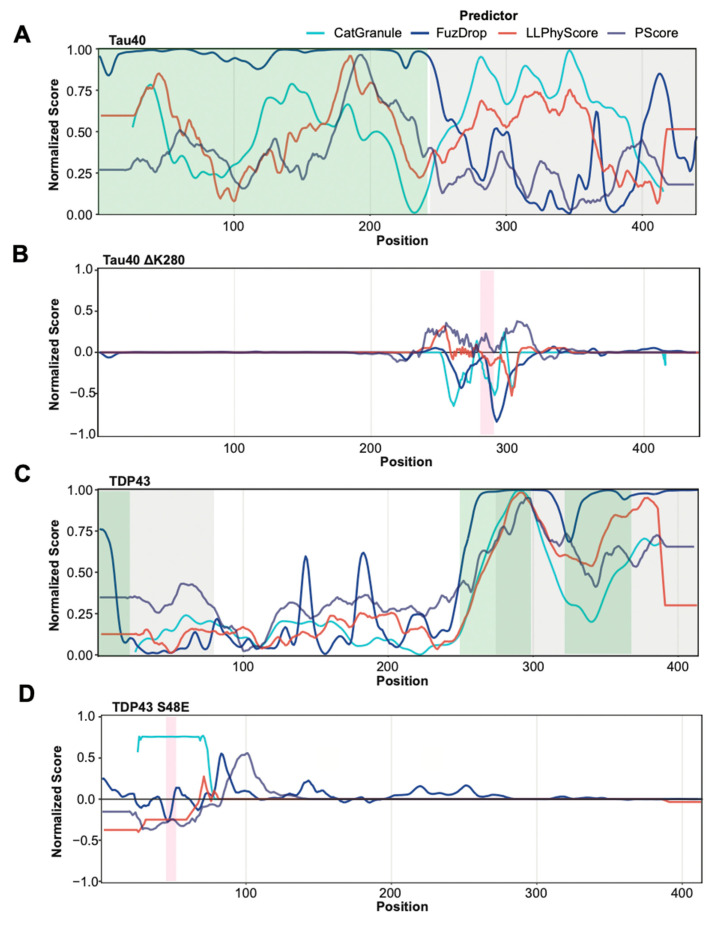
Prediction profiles of selected proteins and mutants. (**A**,**C**) Normalized prediction scores for Tau40 (**A**) and TDP43 (**C**) provided by catGranule, FuzDrop, PScore and LLPhyScore (colour legend on top). Protein segments that have been experimentally identified as being involved in phase separation are highlighted in grey. Protein segments predicted by IUPred2 as disordered are highlighted in green. (**B**,**D**) Normalized delta scores (difference between scores of mutant and wild type sequences) for Tau40 (**B**) and TDP43 (**D**) mutants provided by catGranule, FuzDrop, PScore and LLPhyScore. Mutated residue areas are highlighted in magenta. (**B**) Removal of lysine 280 in Tau40 induces a higher propensity to form beta strands, thus driving oligomerization and phase separation [61]. Most tested methods predict the opposite. (**D**) The S48E mutation in TDP43 abolishes phase separation by blocking the TDP43 dimerization interface [62], which is correctly predicted by most tested methods. Of note, this mutation is in a region not predicted to be involved in phase separation by the tested methods (see (**C**)).

Most predictors were trained on datasets that contain a large number of proteins that phase separate due to their intrinsically disordered regions. Indeed, some are particularly trained to recognize disordered regions that phase separate. Therefore, we tested whether these methods are able to separate disordered regions that have been found to be involved in phase separation from those that were not. To this end, we identified new negative and positive sets by selecting residues from PR and NR that are part of segments of 30 or more contiguous amino acids predicted to be disordered by IUpred2. Using these subsets of annotated regions for prediction assessment provides a less favorable picture (Figure 3B). The performance of all predictors is quite modest, showing only slightly above random classification. Figure 4A,C provide specific examples that illustrate the prediction challenges. Shown are the prediction profiles for the longest isoform of human Tau (Tau40 in A) and for human TDP43 (in C). Tau40 has a long, disordered segment ranging from the N-terminus up to residue 300 (green shaded area in Figure 4A), and all tested methods assign high prediction scores (normalized scores > 0.5) to at least one region in this segment. However, the region identified to phase separate on its own experimentally is at Tau40′s C-terminus (grey shaded area) [58]. TDP43 has disordered segments at the N and C-terminus (green shaded areas in Figure 4C). The ones at the C-terminal end of the protein are part of regions that have been identified to be important to phase separation of this protein and are correctly identified by all methods [59,60]. However, the N-terminal segment that is part of our positive test set is missed by most of them. Overall, our benchmarking suggests that the tested methods struggle in separating disordered regions that participate in phase separation from those that do not. This said, it needs to be stressed that our greedy annotation does not distinguish between those sections of proteins which are necessary and/or sufficient for phase separation. Moreover, experimental testing for phase separation does not always cover the entire protein and tested segments can be very long, which can result in incorrectly assigned residues in our PR and NR sets.

### 3.5. Predicting Mutation Impact on Phase Separation

Robust prediction at the residue level should, in principle, serve to estimate the impact of point mutations on protein phase behavior. Therefore, we examined whether available methods correctly predict the impact of mutations on phase separation in three well-characterized proteins: FUS, Tau40, and TDP43. To this end, we collected 13 mutations from the PhaSepDB database with an annotated impact on protein phase behavior. Based on whether a mutation caused proteins to phase separate at lower or higher concentration in vitro, i.e., altering the saturation concentration, we labelled mutants as phase separation promoting or inhibiting. Figure 4B,D show predictions for two selected mutants in Tau40 and TDP43, illustrating that single amino acid substitutions can affect prediction scores distant from the mutation site. These medium-range effects are likely the result of window-averaged scores calculated with these methods using windows of different length. To determine the effect of a mutation, the score change, defined as the mutant score minus the corresponding wild type score, was calculated. An increase or decrease in likelihood for phase separation was then assessed by first calculating a normalized near-site Z-score. Z-scores above the 95% confidence interval (CI) were then classified as a phase separation promoting mutation. Scores below the 95% CI were considered to be an inhibitory mutation (see Methods). Using this approach, around half of the mutations are correctly classified by most predictors, with PScore and LLPhyScore displaying the best performance (Table 1). The modest performances motivated us to use a more straightforward score interpretation. We simply classified mutants with positive and negative mean-delta scores as phase separation promoting and inhibiting, respectively. The performance did not improve for the tested methods, with the exception of FuzDrop, for which the prediction accuracy came close to the one of the other predictors.

The number of mutations we assessed in this test is small. Moreover, the predictors that we used are not directly trained on saturation concentration data and may thus capture features that may impact phase behavior but are not directly linked to saturation concentrations. Therefore, we extended our list of mutants to include those that affect fluidity or other phase behavior. Specifically, we labelled mutations that decreased the saturation concentration or decreased the fluidity of biological condensates as phase-separation-promoting mutants, while mutations which did the opposite were labelled as inhibitory mutations. Predictors did not perform better on this extended set of mutants, independent of the criteria we used to classify mutants (Table 1, numbers in parenthesis).

Finally, we tested whether there is consistency across the methods tested in their prediction of the effect of amino acid substitutions on phase behavior and whether individual predictors show clear trends for which residues promote phase separation at a given position. To this end, we carried out a computational saturation mutagenesis experiment for S48 of TDP43 (Supplementary Table S2 and Figure 4D). Across all methods, the majority of the substitutions reduce the phase separation prediction score. However, there is little consistency across methods. The predictions for only three substitutions are identical in terms of prediction direction, i.e., whether the mutation enhances or reduces phase separation. Only mutations to isoleucine and tryptophan are consistently predicted to reduce phase separation, while all methods predict glycine to enhance it. In terms of trends for individual predictors suggesting that certain residue types at position 48 of TDP43 clearly enhance or reduce phase separation, again, a mixed picture emerges. While all charged residues are predicted by catGranule to enhance phase separation (note that the S48E mutations has experimentally been shown to reduce phase separation, Figure 4D), no clear trends emerge from the predictions of the other methods tested. This may not come as much of surprise. PScore and LLPhyScore exploit the likelihood of pi–pi and other types of molecular interactions that can involve amino acids with very different physico-chemical properties and depend on whether the amino acid in question is buried or solvent exposed [36]. Overall, the various tests with amino acid substitutions demonstrate that only about half of the tested mutations are correctly classified by currently available tools and that little consistency is seen between predictors, in agreement with the limited performance that we measured on the identification of segments that are involved in phase separation.

## 4. Discussion

Protein phase separation and biomolecular condensate formation is now front-and-center in leading structural and cell biology research. Remarkable insights into the underlying mechanisms and functional roles of this process are rapidly emerging [4,5,62]. A large part of this rapidly growing research field focuses on improving our understanding of the molecular mechanisms that drive the process [51,52,54] and the implementation of gained insights into computational tools that predict protein phase behavior. Here, we assess the performance of several state-of-the art predictors. Our analysis reveals that the predictors tested perform very well in the identification of biomolecular condensate members that act as scaffolds, as well as in the prediction of the likelihood for a protein to undergo phase separation under appropriate conditions in vitro. However, the current method’s performance is much poorer in the identification of biomolecular condensate client proteins, amino acid segments that are involved in phase separation, and the effect that single amino acid substitutions have on phase behavior.

Prediction performances seem paradoxical, as correct predictions for condensate membership of drivers/scaffolds and in vitro phase separation are based on an understanding of the molecular mechanisms that underly phase separation. However, the limited performance when predicting segments that are involved in phase separation or classifying amino acid substitutions according to their effects demonstrates, instead, that the current understanding of the molecular interactions that drive phase separation is insufficient, at least as implemented in most of the tested computational tools. This finding may not be surprising, as we are just at the beginning of dissecting the broadly occurring phenomenon of condensate formation at the molecular level. Not one but multiple “stickers” types have been identified. Stickers can be interaction sites on folded domains, MoRFs and sequence motifs in disordered protein regions or patterns of individual or paired amino acids in disordered protein regions. More importantly, the molecular modes of interaction underlying phase separation are diverse, too; charge–charge, charge–pi, dipole-dipole or pi–pi interactions are among them. A recent theoretical study has shown that hydrophobicity, electrostatics and cation–pi interactions alone are insufficient to reproduce phase separation data for Ddx4 [63], highlighting that various interaction modes need to be considered when modeling and predicting protein phase behavior accurately. Most of the predictors tested in this study do not attempt to capture the complexity of the phenomenon at the molecular level of detail, but are purely phenomenological in nature instead. Exceptions are PScore and LLPhyScore, which, perhaps not surprisingly, perform best in the task of identifying segments involved in phase separation and in classifying mutants according to their impact on phase separation. Moreover, temperature, salt, and other biomolecules, such as RNA, impact phase behavior of proteins and these factors are not or only poorly accounted for by the tested methods. The first successful attempts to include some of these factors in predictions have been published recently [64]. It also needs to be stressed that the currently available data for training and testing computational tools are incomplete, which is also a limiting factor in our evaluation of existing predictors. The data we used in our assessment are likely to contain false negatives and false positives because, in many cases, not all protein regions have been tested for phase separation and those regions tested are often very long. Moreover, phase behavior in vitro may be distinct from that in the cellular environment.

Why do tested methods perform well in the identification of biomolecular condensate scaffolds and in the prediction of in vitro phase separation? Some top ranked predictors such as MaGS exploit protein features, such as RNA-binding and intrinsic disorder, that are highly enriched among currently known members of biomolecular condensates such as stress granules or P-bodies and proteins that phase separate in vitro, respectively. Stress granules and P-bodies, which are presumed RNA storage and processing sites, are among the biomolecular condensates with best charted membership and many, if not most, of the proteins whose phase behavior has been studied in vitro contain large disordered segments such as prion-like domains or other low-complexity regions. Not surprisingly, the features of RNA-binding and protein disorder perform quite well on their own in identifying stress granule scaffolds and proteins that phase separate in vitro. However, it is well-established that not all disordered proteins phase separate under physiological conditions [65] and that even folded proteins can phase separate [9,66,67]. Similarly, not all biomolecular condensates contain RNA and, therefore, RNA-binding proteins. Thus, it is good to question whether there is a bias in the currently available data. Such bias could, at least in part, explain the high performance of many predictors tested here on scaffolds and in vitro phase separation because they have been trained on currently available data. If so, most available methods may be limited in the identification of completely new phase separating proteins that do not display these “classical” features. It is possible that a bias exists, but more tests on larger datasets that become available in the future will be necessary to answer that question. In any case, our findings suggest that results from current prediction methods need to be interpreted with care, particularly when focusing on residue-level information. Alternatively, more sophisticated coarse-grained or all-atom modeling could be used as these approaches can provide accurate insights at the molecular level [68,69,70].

## 5. Conclusions

We demonstrate that state-of-the-art predictors of phase separation perform well in the identification of biomolecular condensate drivers/scaffolds and of proteins that phase separate in vitro but perform much more poorly in spotting protein segments that are involved in phase separation or predicting the impact of amino acid substitutions on phase behavior. Given that the state of knowledge on protein condensates and phase separation phenomena is rapidly changing, some potential shortcomings in the standing framework of models are highlighted. It is clear that larger and more complete datasets are required to train and test the next generation of predictors. Saturation mutagenesis experiments are required that reveal effects of amino acid changes within stickers and spacers on phase behavior, as well as viscoelastic properties of condensates. Importantly, these effects need to be assessed not only for disordered but also ordered protein regions that drive phase separation, and, ideally under varying solution (pH, salt) conditions. Alternatively, or as a complement, coarse-grained models can be used to simulate the impact of large numbers of sequence mutations on phase behavior and thereby generate valuable datasets that can be used, together with the ones collected experimentally, in supervised learning of the next generation of predictors.

## Figures and Tables

**Table 1:** Table with classification results.

Classification of Mutants Using Confidence Intervals				
	catGranule	FuzDrop	LLPhyScore	PScore
PS-reducing mutations	4/6 (4/11)	1/6 (1/11)	5/6 (6/11)	3/6 (7/11)
PS-promoting mutations	2/7 (2/12)	1/7 (1/12)	2/7 (3/12)	5/7 (6/12)
**Classification of Mutants Based on Net Prediction Change**				
	catGranule	FuzDrop	LLPhyScore	PScore
PS-reducing mutations	4/6 (4/11)	2/6 (5/11)	5/6 (6/11)	3/6 (7/11)
PS-promoting mutations	2/7 (2/12)	3/7 (3/12)	2/7 (3/12)	5/7 (6/12)

X/Y shows how many of the mutations with experimental evidence for saturation concentration changes (Y) are classified correctly (X) by each tested method. Numbers for mutations that also affect other aspects of phase separation are shown in parenthesis. PS: Phase separation.

## Data Availability

The data that support the findings of this study are available from the corresponding author upon request.

## Supplementary Materials

The following supporting information can be downloaded at: https://www.mdpi.com/article/10.3390/biom13030527/s1, Figure S1: Performance of state-of-the art methods on biomolecular condensate localization prediction; Figure S2: Performance of state-of-the art methods on prediction of scaffold localization in biomolecular condensates; Figure S3: Performance of state-of-the art methods and simple protein features for in vitro phase separation prediction; Table S1: All positive datasets used in this study; Table S2: Saturation mutagenesis predictions at position 48 of TDP.
