# Supplementary material for: Comparison of Biomolecular Condensate Localization and Protein Phase Separation Predictors
Source: Biomolecules. 2023 Mar 13;13(3):527. doi: 10.3390/biom13030527 (PMC10046894; doi:10.3390/biom13030527)
Supplement: Supplementary file 1 [file biomolecules-13-00527-s001.zip › Supplementary_figures.pdf]

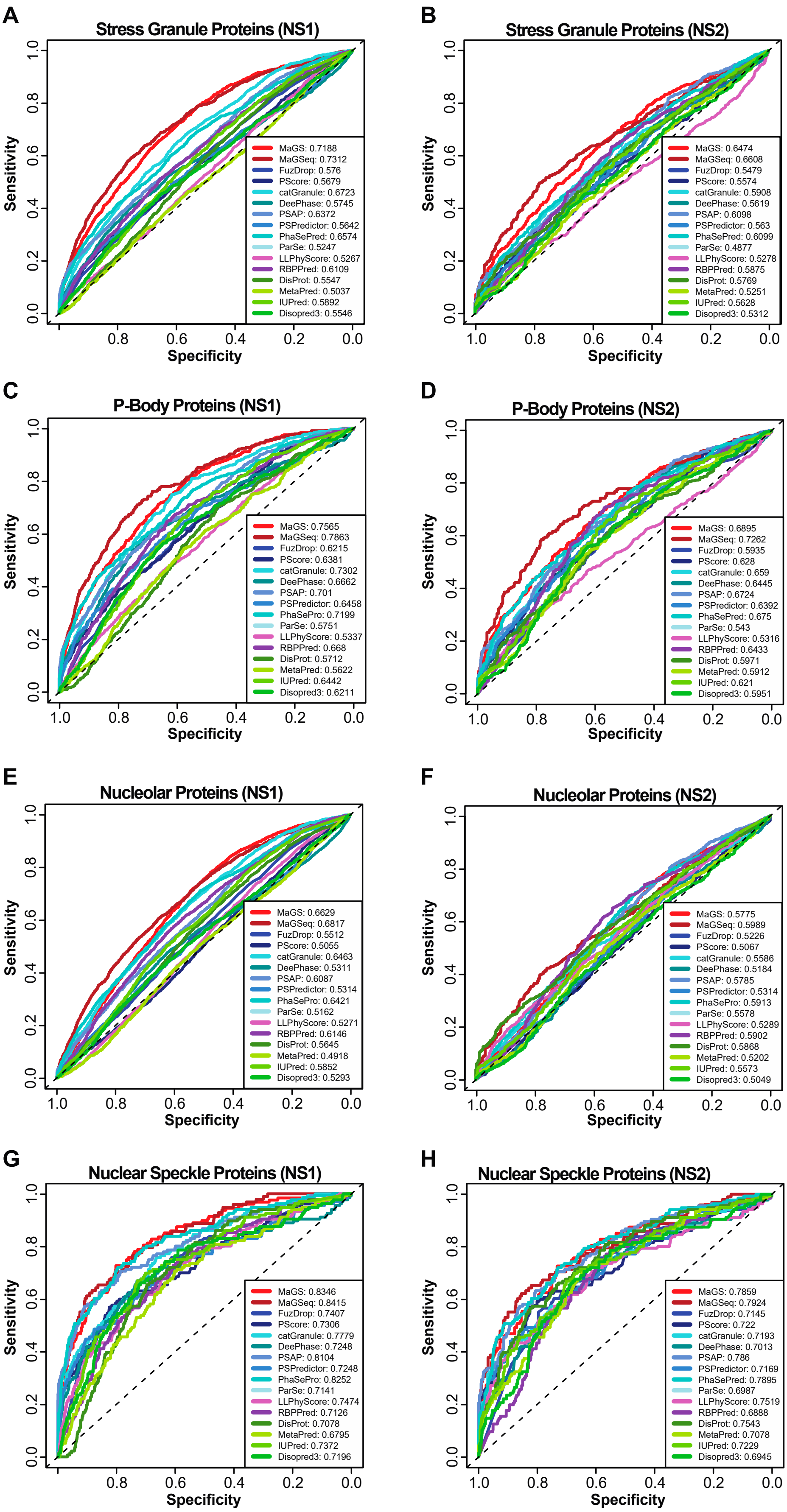

A
Stress Granule Proteins (NS1)
Sensitivity
Specificity
MaGS: 0.7188
MaGSeq: 0.7312
FuzDrop: 0.576
PScore: 0.5679
catGranule: 0.6723
DeePhase: 0.5745
PSAP: 0.6372
PSPredictor: 0.5642
PhaSePred: 0.6574
ParSe: 0.5247
LLPhyScore: 0.5267
RBPPred: 0.6109
DisProt: 0.5547
MetaPred: 0.5037
IUPred: 0.5892
Disopred3: 0.5546
B
Stress Granule Proteins (NS2)
Sensitivity
Specificity
MaGS: 0.6474
MaGSeq: 0.6608
FuzDrop: 0.5479
PScore: 0.5574
catGranule: 0.5908
DeePhase: 0.5619
PSAP: 0.6098
PSPredictor: 0.563
PhaSePred: 0.6099
ParSe: 0.4877
LLPhyScore: 0.5278
RBPPred: 0.5875
DisProt: 0.5769
MetaPred: 0.5251
IUPred: 0.5628
Disopred3: 0.5312
C
P-Body Proteins (NS1)
Sensitivity
Specificity
MaGS: 0.7565
MaGSeq: 0.7863
FuzDrop: 0.6215
PScore: 0.6381
catGranule: 0.7302
DeePhase: 0.6662
PSAP: 0.701
PSPredictor: 0.6458
PhaSePro: 0.7199
ParSe: 0.5751
LLPhyScore: 0.5337
RBPPred: 0.668
DisProt: 0.5712
MetaPred: 0.5622
IUPred: 0.6442
Disopred3: 0.6211
D
P-Body Proteins (NS2)
Sensitivity
Specificity
MaGS: 0.6895
MaGSeq: 0.7262
FuzDrop: 0.5935
PScore: 0.628
catGranule: 0.659
DeePhase: 0.6445
PSAP: 0.6724
PSPredictor: 0.6392
PhaSePred: 0.675
ParSe: 0.543
LLPhyScore: 0.5316
RBPPred: 0.6433
DisProt: 0.5971
MetaPred: 0.5912
IUPred: 0.621
Disopred3: 0.5951
E
Nucleolar Proteins (NS1)
Sensitivity
Specificity
MaGS: 0.6629
MaGSeq: 0.6817
FuzDrop: 0.5512
PScore: 0.5055
catGranule: 0.6463
DeePhase: 0.5311
PSAP: 0.6087
PSPredictor: 0.5314
PhaSePro: 0.6421
ParSe: 0.5162
LLPhyScore: 0.5271
RBPPred: 0.6146
DisProt: 0.5645
MetaPred: 0.4918
IUPred: 0.5852
Disopred3: 0.5293
F
Nucleolar Proteins (NS2)
Sensitivity
Specificity
MaGS: 0.5775
MaGSeq: 0.5989
FuzDrop: 0.5226
PScore: 0.5067
catGranule: 0.5586
DeePhase: 0.5184
PSAP: 0.5785
PSPredictor: 0.5314
PhaSePro: 0.5913
ParSe: 0.5578
LLPhyScore: 0.5289
RBPPred: 0.5902
DisProt: 0.5868
MetaPred: 0.5202
IUPred: 0.5573
Disopred3: 0.5049
G
Nuclear Speckle Proteins (NS1)
Sensitivity
Specificity
MaGS: 0.8346
MaGSeq: 0.8415
FuzDrop: 0.7407
PScore: 0.7306
catGranule: 0.7779
DeePhase: 0.7248
PSAP: 0.8104
PSPredictor: 0.7248
PhaSePro: 0.8252
ParSe: 0.7141
LLPhyScore: 0.7474
RBPPred: 0.7126
DisProt: 0.7078
MetaPred: 0.6795
IUPred: 0.7372
Disopred3: 0.7196
H
Nuclear Speckle Proteins (NS2)
Sensitivity
Specificity
MaGS: 0.7859
MaGSeq: 0.7924
FuzDrop: 0.7145
PScore: 0.722
catGranule: 0.7193
DeePhase: 0.7013
PSAP: 0.786
PSPredictor: 0.7169
PhaSePred: 0.7895
ParSe: 0.6987
LLPhyScore: 0.7519
RBPPred: 0.6888
DisProt: 0.7543
MetaPred: 0.7078
IUPred: 0.7229
Disopred3: 0.6945

**Figure S1: Performance of state-of-the art methods on biomolecular condensate localization prediction**

(A-H) ROC curves calculated with the predictions by catGranule, DeepPhase, FuzDrop, MaGS, MaGSeq, ParSe, PSAP, PScore, PSPred and LLPhyScore, as well as with simple protein features calculated by the methods RBPPred, DisProt, MetaPred, IUPred, and Disopred3. Curves are calculated using all proteins of indicated cellular condensates as positives and NS1 (A, C, E, G) or NS2 (B, D, F, H) as negative set. AUC values are provided for each method in the legend.

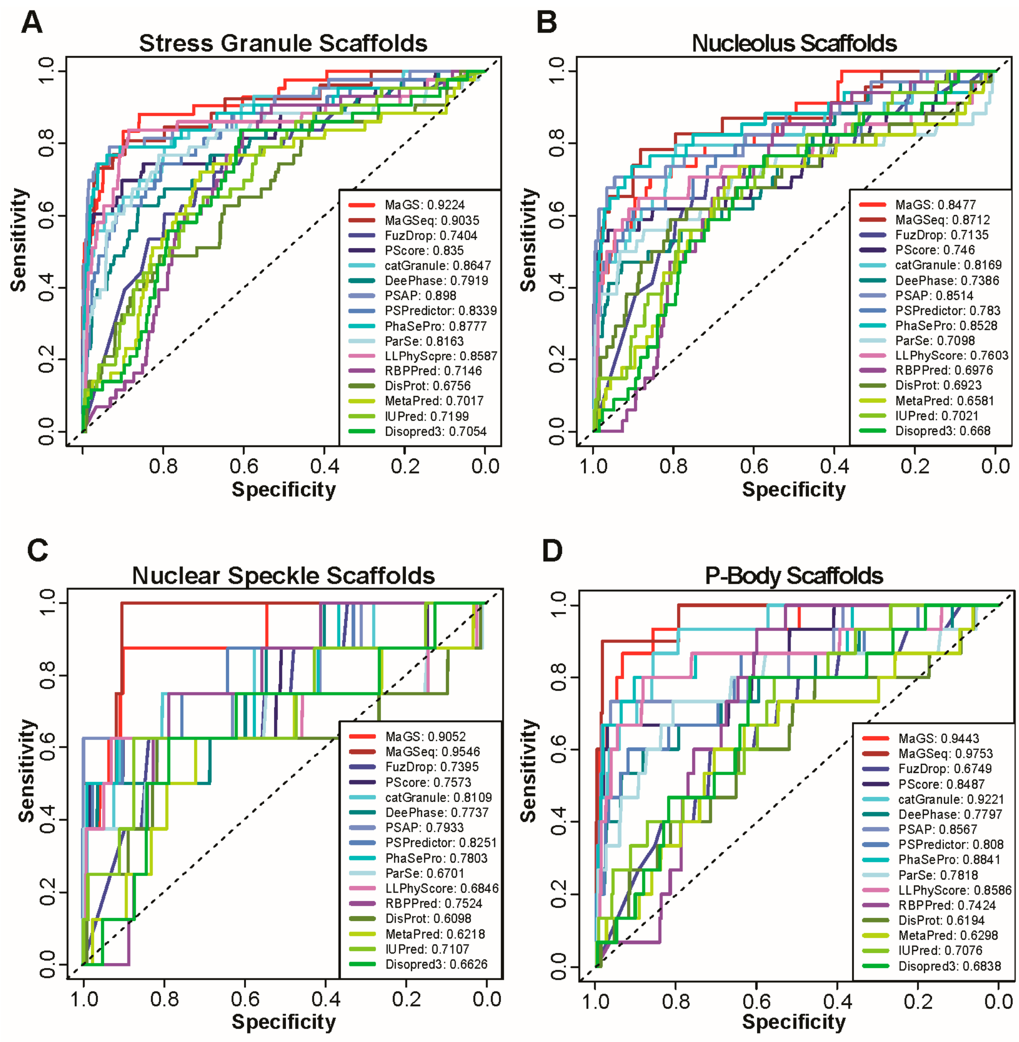


**Figure S2: Performance of state-of-the art methods on prediction of scaffold localization in biomolecular condensates**

(A to D) ROC curves calculated with predictions by the same methods used as Supplementary Figure S1. Curves are calculated using known scaffolds of stress granules (A), nucleolar condensates (B), nuclear speckles (C), or P-bodies (D) as positives, while NS1 is used as negative set. AUC values are provided for each method in the legend.

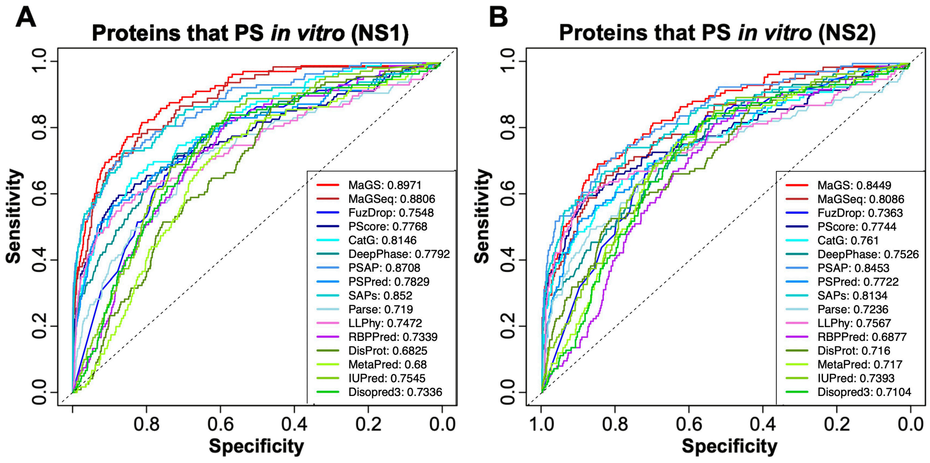


**Figure S3: Performance of state-of-the art methods and simple protein features for *in vitro* phase separation prediction**

(A and B) ROC curves calculated with predictions by the same methods used for Supplementary Figure S1. Curves are calculated using proteins known to phase separate *in vitro* as positives and NS1 (A) or NS2 (B) as negative set. AUC values are provided for each method in the legend
